# North West London New Model of Care Project (NMOC) – improving inpatient mental health care for children and young people

**DOI:** 10.1192/bjo.2021.535

**Published:** 2021-06-18

**Authors:** Jovanka Tolmac, Alun Lewis, Azer Mohammed, Elizabeth Fellow-Smith, Johan Redelinghuys, Braulio Girelas

**Affiliations:** 1 Consultant Child and Adolescent Psychiatrist, Harrow CAMHS, Central and North West London NHS Foundation Trust; 2 NMOC Project manager, West London NHS Trust; 3 Consultant Child and Adolescent Psychiatrist, Westminster CAMHS, Central and North West London NHS Foundation Trust; 4 Consultant Child and Adolescent Psychiatrist, West London NHS Trust; 5 CAMHS Clinical Director, West London Trust; 6 Clinical Research Fellow, Centre for Psychiatry, Imperial College, Centre for Paediatrics and Child Health, Faculty of Medicine, Imperial College, Honorary Spr, Harrow CAMHS, Central and North West London NHS Foundation Trust

## Abstract

**Aims:**

Specialised inpatient mental health services for children and young people are commissioned and managed by NHS England (NHSE) and provided by NHS as well as independent sector. The access to beds has been managed nationally with young people admitted far from home. There were capacity issues identified in London. To address these concerns, NHSE invited organisations to work in partnership to co-design and establish new models of care. This is one of the first of such projects, set up to manage the budget for children and young people's beds on behalf of NHSE and change the way of managing and monitoring admissions.

Our aims:

To reduce length of inpatient stay

To enable admission of young people as close to home as possible

To improve resource efficiency, capacity and capability of managing young people in crisis in the community.

**Method:**

A number of changes were introduced, including engagement of community and inpatient clinical staff, repatriation to units closer to home and introduction of CRAFT meetings (early review meetings in inpatient units to enable timely and effective discharge planning and support back to local services). The implementation has been closely monitored by the project manager and clinical group, which included representatives from all organisations involved.

**Result:**

After four years, young people are admitted to hospitals closer to home and the length of inpatient stay has decreased by 18%. The number of admissions has decreased by 28%. Out of area occupied beds days have been decreased by 66%.

Significant recurrent budget savings have been achieved. Over the past three years, these savings have been reinvested in developing crisis community support and more specialist community services within CNWL and West London Trust.

**Conclusion:**

There have been considerable benefits of multiple organisations working in partnership to improve patients care. The success of the project has created further opportunities for the development of services which provide safe and effective alternatives to admission (such as crisis services, home treatment teams and specialized community services). In summary, this collaborative model has improved the quality of care and experience for young people and reduced the need for psychiatric admission.

